# Laboratory and experimental hut trial evaluation of VECTRON ^™^ T500 for indoor residual spraying (IRS) against insecticide resistant malaria vectors in Burkina Faso

**DOI:** 10.12688/gatesopenres.13578.2

**Published:** 2022-07-25

**Authors:** Koama Bayili, Hyacinthe D. Ki, Bazoma Bayili, Bazoumana Sow, Abdoulaye Ouattara, Graham Small, Aristide S. Hien, Roch K. Dabire, Abdoulaye Diabate

**Affiliations:** 1 Entomologist, Institut de Recherche en Sciences de la Santé, Bobo-dioulasso, 545, Burkina Faso; 2 Entomologist, Université Nazi Boni, Bobo-Dioulasso, Burkina Faso; 3 Senior Technical Manager, Innovative Vector Control Consortium, Liverpool, Liverpool L3 5QA, UK; 4 Institut de Recherche en Sciences de la Santé, Bobo-Dioulasso, Burkina Faso

**Keywords:** Malaria, Anopheles gambiae, VECTRON™ T500, Insecticide Residual Spray (IRS), Pyrethroid resistance, Residual efficacy

## Abstract

**Background:** Malaria cases in some areas could be attributed to vector resistant to the insecticide. World Health Organization recommended insecticides for vector control are limited in number. It is essential to find rotational partners for existing Indoor Residual Spraying (IRS) products. VECTRON ^™^ T500 is a novel insecticide with broflanilide as active ingredient. It has a mode of action on mosquitoes completely different to usually used. The aim of this study was to determine the optimum effective dose and efficacy of VECTRON ^TM^ T500 against susceptible and resistant strains of *Anopheles* in Burkina Faso.

**Methods: **VECTRON™T500 was sprayed at 50, 100 and 200 mg/m² doses onto mud and concrete blocks using Potter Spray Tower. The residual activity of broflanilide was assessed through cone bioassays 1 week and then monthly up to 14 months post spraying. Its efficacy was evaluated at 100 and 150 mg/m² against wild free-flying mosquitoes in experimental huts on both substrates. Actellic 300CS was applied at 1000 mg/m² as reference product. Cone assays were conducted monthly, using susceptible and resistant mosquito strains.

**Results: **In the laboratory, VECTRON ^™^ T500 showed residual efficacy (≥80% mortality) on *An. gambiae *Kisumu up to 12 and 14 months, respectively, on concrete and mud blocks. Similar results were found with 100 and 200 mg/m² using *An. coluzzii *pyrethroid resistant strain. In experimental huts, a total of 19,552 *An. gambiae *s.l. were collected. Deterrence, blood-feeding inhibition and exophily with VECTRON™ treated huts were very low. At 100 and 150 mg/m², mortality of wild *An. gambiae *s.l. ranged between 55% and 73%. Monthly cone bioassay mortality remained >80% up to 9 months.

**Conclusions: **VECTRON™ T500 shows great potential as IRS formulation for malaria vector control. It can be added to the arsenal of IRS products for use in rotations to control malaria and manage mosquito insecticide resistance.

## Introduction

Malaria remains one of the most critical public health problems in Africa, despite intense national and international efforts to control it. According to the World Health Organization (WHO), malaria caused 409,000 deaths out of 229 million cases registered in 2019 ^1^. A parasitic disease, malaria is caused by a hematophagous protozoan of the genus *Plasmodium*. This pathogen is transmitted to humans during the bite of an infected *Anopheles* female mosquito. Current measures to control malaria are based on early detection and appropriate treatment of malaria cases and malaria vector control. Vector control is based mainly on the use of Long-Lasting Insecticidal Nets (LLINs) and indoor residual spraying (IRS) ^2^, which aims to reduce vector densities and human-vector contact. Widespread deployment of LLINs and IRS by countries has played a crucial role in the reduction of malaria incidence and mortality in sub-Saharan Africa in the last 20 years ^1, 3^. It was estimated that 1.5 billion malaria cases and 7.6 million malaria deaths have been averted during the period of 2000 to 2019 ^1^ due to malaria control policy put in place by countries. These policies include mass distribution of insecticide-treated nets, mass use of IRS, prompt malaria cases management, and use of drugs to prevent malaria.

Indoor residual spraying (IRS) is one of the main vector control methods used for preventing malaria in many malaria-endemic countries ^2^. IRS can reduce malaria transmission by reducing female mosquito density and longevity when the IRS product is applied inside residential houses. The residual insecticide on the potential resting surfaces such as internal walls, eaves and ceilings is effective against female mosquitoes that contact these surfaces and are killed ^4, 5^. Historically, IRS was the principal tool of the global malaria eradication campaign that allowed malaria elimination from Europe and several countries in the Americas and the Caribbean during the 1950s and 1960s ^4^. Reduction in malaria morbidity and mortality was observed in endemic countries in Africa and Asia that increased significantly the coverage of IRS during the last 20 years ^5^. Unfortunately, the success of malaria control programs is being compromised by the emergence and spread of insecticide resistance in major mosquito vector species ^6– 10^. This has led in recent years to the combination of IRS and LLIN in some African countries to increase the impact of vector control ^11^. The Global Plan for Insecticide Resistance Management (GPIRM) has recommended a rotation of non-pyrethroid insecticides with different modes of action for IRS in countries where IRS and LLINs are combined ^12^. Only two non-pyrethroid insecticides are currently listed by WHO Prequalification Unit Vector Control Product Assessment Team (WHO PQT/VCP) as IRS formulation products. They are clothianidin (a neonicotinoid insecticide; available as SumiShield 50WG and its coformulated with deltamethrin as Fludora Fusion) and pirimiphos-methyl (an organophosphorus insecticide formulated as Actellic 300CS) ^13^. However, to properly implement an insecticide resistance management strategy based on the rotation of insecticides with different modes of action, IRS products containing at least 3 different insecticides will be required. Therefore, finding additional alternative insecticides with novel modes of action to vector control has become a priority ^14^. VECTRON™ T500, containing the active ingredient broflanilide ( *N*-[2-bromo-4-(perfluoropropan-2-yl)-6-(trifluoromethyl)phenyl]-2-fluoro-3-( *N*-methylbenzamido)benzamide]), is a novel insecticide formulation developed by Mitsui Chemicals Agro, Inc., (MCAG; Tokyo, Japan) for IRS use to control malaria vectors or other pests ^15^. It has the potential to control mosquitoes that have become resistant to pyrethroids and other known classes of conventional insecticides ^16^. Broflanilide has been categorized as a member of a new group, Group 30: GABA-gated chloride channel allosteric modulators, by the Insecticide Resistance Action Committee (IRAC). It targets the GABA-receptor of chloride channels in the nervous system of insects ^17^. Broflanilide is a meta-diamide insecticide that has a distinct mode of action compared to conventional insecticides currently used in public health ^18^. It has a lower action on the mosquitoes than pyrethroids insecticide. There is currently no known cross-resistance to broflanilide via mechanisms of resistance to other public health insecticides. It has also shown low acute toxicity to non-target aquatic organisms ^19^, which demonstrates its high potential for use in public health and agriculture.

Before new vector control products can be introduced to the market, the optimal dose and formulation of the active ingredient must be determined. In addition, the residual efficacy of this dose must be evaluated against the target mosquitoes. It is in this context that this study aimed to determine the dose and efficacy, including the residual activity, of this new product, VECTRON™ T500, which is a wettable powder containing 50% broflanilide (w/w) as an active substance. VECTRON™ T500 was tested against susceptible and resistant strains of *Anopheles* malaria vectors in Burkina Faso. Firstly, a a laboratory study was conducted using blocks made of different substrates, to determine the most suitable doses for field trials. Secondly, VECTRON™ T500 was tested at two application rates in an experimental hut trial using two different wall substrates, mud and concrete, to assess its efficacy against free flying mosquitoes following WHO guidelines.

## Methods

### Study area and mosquitoes

The laboratory study was conducted at the IRSS (Institut de Recherche en Sciences de la Santé) test facility in Burkina Faso under standard environmental conditions (27±2 °C and 75±10% relative humidity (RH)). The experimental hut trial was conducted at the field station in Vallée du Kou, an irrigated rice field area developed in 1970. The site is characterized by wooded savannah and covers 1,200 ha between 4˚24’59’’ longitude west and 11˚24’ latitude and contains seven discrete villages. Mean annual rainfall is about 1,100 mm and rice is the major crop. Few insecticides are used on this crop, but they are widely used in the surrounding villages for cotton cultivation. Thanks to irrigation, the plain provides mosquitoes with permanent, sunny, and nutrient-rich breeding sites for the development of *Anopheles* larvae. Mosquitoes are found year-round, but the peak density is observed in August to September during the rainy season. *An. coluzzii* is predominant throughout the year ^20^ and is highly resistant to pyrethroids and dichlorodiphenyltrichloroethane (DDT) ( *kdr* frequency: 0.8-0.95), with a rise in *ace-1* frequency also being observed ^21– 23^. Mechanisms of metabolic resistance, such as cytochrome P450s, esterases and also non-detoxification genes have been detected ^8, 10^. The presence of multiple resistance genes in the mosquito populations makes this area an ideal site to evaluate the effectiveness of new insecticides against mosquitoes that are resistant to conventional insecticides.

### Laboratory study

***Preparation and treatment of block substrates***: Two types of substrates were used to prepare IRS blocks for laboratory tests. Mud blocks were made by mixing 100g of mud and 25ml of water. The mud was from the experimental hut study site (Bama; 4°24’59” longitude west; 11°24’ latitude) to minimize variation between the mud used in laboratory and experimental hut trials. Concrete blocks were made by mixing 33g cement, 66g sand and 20mL water. Blocks were shaped in Petri dishes (9 cm diameter and 1 cm thick). Mud blocks and concrete blocks were left to dry for a minimum of 1 week and for 1 month, respectively, at 27 °C ± 2 °C and 75% ± 10% relative humidity before insecticide was applied. The pH of the concrete blocks was tested on the day they were to be sprayed by scraping 5g of concrete from a block, adding 15ml distilled water, mixing thoroughly, and measuring with a pH meter (HANNA Instruments, model Hi 9813-5): blocks with a pH between 6-10 (mud and concrete) were judged suitable for use. The blocks were sprayed with the different treatments ( Table 1) using a homogeneous solution of each dose. The VECTRON™ T500 product as batch no 18I-3671 was provided by Mitsui Chemicals Agro, Inc. (MCAG). Spraying was done using a calibrated Potter Precision Laboratory Spray Tower (Burkard Manufacturing Co Ltd, Rickmansworth, UK) which is internationally recognized as the most precise method of chemical spraying in the laboratory as described in WHO testing guidelines ^24^. All treated blocks were stored at 30 °C ± 2 °C and 75% ± 10% RH in between bioassays. In total, five blocks of each substrate type were prepared and sprayed for each dose.

**Table 1.** Substrates and treatments used for laboratory studies.

Treatments	Application rates of treatments	Substrates	Number of blocks
VECTRON™ T500	50 mg a.i./m ^2^	Concrete	05
VECTRON™ T500	100 mg a.i./m ^2^	Concrete	05
VECTRON™ T500	200 mg a.i./m ^2^	Concrete	05
Negative control	Distilled water	Concrete	05
VECTRON™ T500	50 mg a.i./m ^2^	Mud	05
VECTRON™ T500	100 mg a.i./m ^2^	Mud	05
VECTRON™ T500	200 mg a.i./m ^2^	Mud	05
Negative control	Distilled water	Mud	05

***Residual efficacy of broflanilide WP (VECTRON™ T500) in laboratory cone bioassays***: After spraying, WHO cone bioassays were performed according to WHO guidelines ^24^ to evaluate the residual activity of insecticide on the substrates. Bioassays were performed at 1 week and then monthly up to 14 months post spraying, by attaching the cones to the treated and control blocks. For each insecticide dose and substrate type used, 100 unfed female mosquitoes aged 2 to 5 days were exposed in WHO polyvinyl chloride cones (obtained from the Vector Control Research Unit (VCRU) WHO Collaborating Centre, Universiti Sains Malaysia, Penang, Malaysia) for 30 minutes contact time with 10 mosquitoes per cone per block. Two cone bioassays were performed per block, with five blocks of each treatment. *An. gambiae* Kisumu susceptible strain and *An. coluzzii* VK laboratory resistant strain, reared at the IRSS insectary under standard controlled conditions (27±2°C and 75±10% relative humidity), were used. After removal from cones, mosquitoes were transferred into holding cups, provided access to 10% sucrose soaked cotton wool, and held under the same conditions described earlier. Mortality was recorded at 24 hours, 48 hours and 72 hours post exposure in cones.

### Experimental hut trial

***Design of huts***: The experimental huts used were of the West African design ^25^. An experimental hut is a simulated house in which all entering, exiting (exophily), dead and blood fed mosquitoes can be recorded. It is made of local material and is characterized by the presence of a gutter or moat around the hut to protect against ants which would eat dead mosquitoes. It is also characterized by the presence of veranda traps to catch mosquitoes which may exit during the night due to either behavioural or insecticidal effects. Mosquitoes can enter through four window slits constructed from pieces of metal, fixed to create a funnel of 1 cm wide gap to inhibit mosquitoes from exiting. The ceiling of the huts was made of plastic. For interior wall surfaces, two types of material were used: concrete and mud.

***Treatments***: VECTRON™ T500 was evaluated at two application rates (100 mg a.i./m ^2^ and 150 mg a.i./m ^2^ rather than 200mg/m ^2^) tested in laboratory) on concrete and mud walls in the experimental huts. The reference product was Actellic® 300CS (Syngenta), which contains the organophosphate insecticide pirimiphos-methyl as the active ingredient, It was used at the recommended dose of 1000 mg a.i./m ^2^. A negative control, sprayed only with distilled water, was also included. Table 2 below summarises the different treatment arms and substrates that were tested.

**Table 2.** Treatments and substrates used for the experimental hut study.

Treatment	Application rates with respect to the active ingredient	Walls	Number of huts
**VECTRON™ T500**	100 mg a.i./m ^2^	Concrete	01
**VECTRON™ T500**	150 mg a.i./m ^2^	Concrete	01
**VECTRON™ T500**	100 mg a.i./m ^2^	Mud	01
**VECTRON™ T500**	150 mg a.i./m ^2^	Mud	01
**Positive control** **(Actellic® 300CS)**	1000 mg a.i./m ^2^	Concrete	01
**Negative control**	Distilled water	Concrete	01

***Insecticide application***: The IRS treatments were applied at the specified dosages ( Table 2) to the internal walls of experimental huts and the hut ceiling using a MICRON CS-10 10L compression sprayer, fitted with a red 4.2 bar CFV and a T-Jet 8002E flat fan nozzle. The target volume ejected was 560 mL/min. All sprayers were calibrated with water prior to treatment of huts. All sprayers were equipped with pressure gauges, and initial pressure settings were conducted at 60 psi for consistency. The huts were prepared before spraying by marking swaths on the walls and ceiling, each swath being 75 cm in width and with a 5 cm overlap with the next swath. The safety precautions, mixing, handling, spray techniques and spray tank washing were all done according to standard procedures as outlined in the WHO manual for IRS. Prior to spraying, the spray operator practiced several times on blank walls using a tank filled with water to ensure that a constant flow rate was obtained before treatment started. A digital metronome (freeware from Metronome Beats v. 2.3.3, Stonekick 2013) synchronized with a digital stopwatch was used to enhance the consistency of applications (6 seconds per spray swath). The use of the digital metronome provided an audible guide to spray operators. An orientation pole was attached to the handle of the sprayer during spraying to maintain the correct distance of the sprayer nozzle from the walls. It was attached to the handle of the sprayer during spraying. Five labelled filter papers (Whatman™ No. 1 10cm × 10cm) were fixed onto the four walls and ceiling of the huts. The filter papers were removed after spraying, dried, grouped by hut and treatment, and carefully packed in aluminium foil for subsequent High performance Liquid Chromatography (HPLC) analysis at the Liverpool School for Tropical Medicine (LSTM), to provide a measure of the quality of the treatment applications. Insecticides were mixed homogeneously in the spray tank. Spraying was done alternately from roof to floor and then from floor to roof to treat each hut. After spraying the wall, the tarpaulin ceiling previously arranged on a plastic support was also sprayed. The sprayer tank was shaken frequently to ensure proper mixing. After spraying of each treatment, the solution remaining in the pump was removed and the volume measured. This measurement made it possible to determine the actual quantity of treatment solution applied per hut.

***Trial procedure***: Evaluation of free flying mosquitoes started five days after applying the treatments inside the 6 huts. Cows were used as bait for mosquito attraction in place of human volunteers as the local *An. coluzzii* population is relatively zoophilic (according to unpublish previous trials data) and also the toxicity, and potential risk of the VECTRON™ T500 product had not been fully assessed at the time of study initiation. In total, 14 cows, male and female, aged between 2 and 3 years, were purchased locally. A veterinarian was recruited to follow their health. They were used in this study according to his requirements to ensure their good health. Cows were divided in two groups (7 per group): one group (6 cows and 1 on back up) was used during one week and the second during the following week. The cows of each group were randomized on the first day of use and placed inside the huts in containment crates made of wood. Cows were rotated between huts each night according to a Latin square design to control for any variation in the attractiveness of individual cows to the mosquitoes. Thus, every cow spent one night in each hut during the round of 6 nights. They were placed inside huts at dusk (7:00 pm) and remained inside until dawn.

Each morning, volunteers, recruited from the village around the huts station and trained in mosquito collection, entered the huts to collect the mosquitoes that had entered overnight. Dead and live mosquitoes were collected from the floor, the walls and the ceiling of the hut and from the veranda trap, and placed into collection tubes. Mosquitoes were put in different bags for each collection compartment and transferred to the laboratory. Species identifications were made using the appropriate taxonomic keys. Mosquitoes were scored by location as dead or alive and as fed or unfed. Live mosquitoes were placed in cups covered with clean netting and provided with a 10% glucose solution for assessment of delayed mortality up to 72 hours after collection. Trial was run on 12 rounds of 6 days each.

The main outcomes measured were:

- Deterrence: reduction in treated hut mosquito entry rates relative to the negative control hut;
- Induced exophily: proportion of mosquitoes that exit early and are found in exit traps;
- Blood-feeding inhibition: the reduction in blood feeding of mosquitoes compared with those in the negative control huts;
- Immediate and delayed mortality: proportion of mosquitoes that are found killed early morning and after 72 hours of holding.

***Evaluation of insecticide residual activity using cone tests***: Residual activity of the different treatments was assessed at 1 week and then monthly after spraying up to 9 months for the VECTRON™ T500 100 mg a.i./m² and Actellic® 300CS treatments and up to 12 months for the VECTRON™ T500 150 mg a.i./m² treatment. Females of the susceptible strain *An. gambiae* Kisumu strain and the resistant strain *An. coluzzii* VK strain were tested using WHO standard cone bioassays on the treated walls and ceiling ^24^. Two cones were attached with masking tape on each of the hut inner walls (the four walls and the ceiling) to obtain ten (10) cones per hut. Ten (10) females were exposed in each cone by plugging the cone with cotton wool after the introduction of mosquitoes. After 30 minutes of contact, mosquitoes were removed and placed in 150-ml plastic cups with access to the glucose solution (10%) provided via cotton wool. Mosquitoes were quickly transferred to the holding room in Bobo-Dioulasso (30 min drive) and maintained at a temperature of 27°C ± 2°C and 75% ± 10% RH. Knockdown was recorded 60 minutes after exposure, and mortality was recorded 24, 48 and 72 hours after exposure in cones.

### Insecticide application quality

The aim of this chemical analysis was to assess the quality of the spraying by comparing the doses of insecticides on the papers with the target doses. The difference of the two doses in percentage should be in the range of ±50% of the target dose according to WHO recommendation ^26^.

The five labelled filter papers (10 cm × 10 cm) fixed to the walls and ceiling of each hut before spraying were removed after spraying, packed in aluminium foil separately, and put in labelled bags. The packed samples were stored in a refrigerator at +4°C temperature and were then shipped to LSTM/LITE in Liverpool for HPLC analysis.

Broflanilide content was determined by reversed-phase high-performance liquid chromatography (HPLC) using UV detection at 226 nm and dicyclohexyl phthalate (DCP) standard as an internal standard. Briefly, a hole punch (0.635cm radius) was used to cut 12 circles from each filter paper. The pieces of each filter were placed into a glass tube and 5 ml of extraction solution was added, consisting of 100 µg/ml of DCP in methanol. The glass tubes were capped with tin foil and a screw cap and placed into a water bath sonicator. Samples were sonicated for 60 minutes at room temperature. Once sonication was completed, a syringe and PTFE filter (0.2 µm) was used to transfer 1 ml of each solution to a 1.5 ml Eppendorf tube. Using a 200µl micropipette, a 100µl aliquot of each sample was pippeted into a labelled HPLC vial. The HPLC was equipped with a detector suitable for operation at 226 nm, a constant temperature column compartment and an injector capable of delivering 20 µl injection volume. A Thermo Scientific Hypersil Gold column (Thermo Fisher Scientific, U.K.; Particle size: 5µm) was attached to the HLPC equipment. The mobile phase was acetonitrile and water mixed in a 7:3 ratio (v/v) with a flow rate of 1 ml/min and detection at 226 nm. The column temperature was between 23°C and 25°C.

### Supplementary tests: : Polymerase Chain Reactions (PCR) and WHO resistance assay

A sub-sample of mosquitoes (610 individual mosquitoes) collected in treated huts were submitted for polymerase chain reaction (PCR) analysis to determine species and the presence of *kdr* resistance by genotyping. The cycling conditions were 10' [30",30",60"] 35c @ 54°C for Sine and 3' [30", 30", 10"] 35c @ 55°C for Kdr_w. The reagents and kits (details in supplementary file 11, *Extended data* ^27^) used are Pool Master Mix, Primers, sterile water, Trizma base, boric acid, EDTA, Agarose Multi, Purpose Agarose, Hexadecyltrimethylammonium bromide, sodium chloride, Trizma hydrochloride solution Ph 8.0, 1M and Ethylenediaminetetraacetic acid, 0,5 M aq. Soln, pH 8.0 Liquid. The primers were: S200X 6.1F: TCG-CCT-TAG-ACC-TTG-CGT-TA, S200X 6.1R: CGC-TTC-AAG-AAT-TCG-AGA-TAC, Kdr_w D1: ATA-GAT-TCC-CCG-ACC-ATG, Kdr_w D2: AGA-CAA-GGA-TGA-TGA-ACC, Kdr_w D3: AAT-TTG-CAT-TAC-TTA-CGA-CA and Kdr_w D4: CTG-TAG-TGA-TAG-GAA-ATT-TA. The main equipments were composed of by thermocyclers (Eppendorf, Biorad, Applied Biosystems), transluminator, migration cuve (Fisherbrand, Apelex), vortex, centrifuges, Eppendorf pipettes, Electrophoresis Power Supply (E 815 CNSort and E 844 CNSort) (details in supplementary file 11, *Extended data* ^27^). The species identification to identify *An. gambiae* complex species used the standard protocol ^28^ and the presence/absence of kdr mutation L1014F (kdr-w) was determined using the protocol described by Martinez-Torres *et al.* ^29^. Mosquitoes were identified as *An. arabiensis*, *An. gambiae* sensu stricto or *An. coluzzii*. For the resistance assays, mosquitoes were classified as SS, RS or RR i.e., homozygous susceptible, heterozygous, or homozygous resistant for the kdr mutation L1014F.

Phenotypic resistance was evaluated using 2 to 5 -day old adult female mosquitoes according to the standard WHO susceptibility test method ^30^. The insecticide and PBO treated papers (impregnated papers) were obtained from WHO laboratory in Malaysia.

### Data management and statistical analysis

WHO cone bioassay and experimental hut trial data were entered using EpiData v 3.1 software (RRID:SCR_008485). Mortality was calculated from the total number of mosquitoes tested per period. If the mortality of the negative control in WHO cone bioassay was between 5% and 20%; mortality of treated mosquitoes was corrected using Abbott’s formula.

**Abbott’s formula**: $\text{Corrected}\;\text{mortality}=\frac{\text{(\%}\;\text{treated}\;\text{mortality}-\text{\%}\;\text{negative}\;\text{control}\;\text{mortality)}}{100-\text{\%}\;\text{negative}\;\text{control}\;\text{mortality}}\times 100$

If negative control mortality was above 20% at 24 hours after exposure, the test data for that day was discarded, and the cone bioassays repeated. In the experimental hut trial, the free flying mosquito data such as the number of mosquitoes that entered, exited, dead inside the hut. or during 72 hours observation time were recorded. The number that succeeded in blood feeding on the cows were calculated for each treatment by compiling the data collected over 12 weeks. The main analyses were performed using R statistical software (RRID:SCR_001905) version 4.1.0 with a significance level of 0.05 for rejecting the null hypothesis following a predefined analysis plan. Mixed effect logistic regression model analysis was conducted using the lme4 package, to compare proportional data by taking mosquitoes exited, blood fed and dead (total mortality) as dependent variables and treatment as categorical covariates (fixed effect), sleepers (cows) and months of the trial (random effect). For overall comparison, the negative control (untreated hut) was kept as a reference category. The primary criteria in the evaluation were blood feeding inhibition and 72 hours mortality. All graphs were produced using Excel 2016.

### Ethical considerations

Institutional ethical approval for the study was obtained on October 2016 from the Institutional (Institut de Recherche en Sciences de la Santé) Ethics Committee for Health Sciences Research (N/Réf. 023- 2016/CEIRES). The cows used in experimental huts to attract mosquitoes were maintained according to the institution's recommendations. Care was taken that the cows were not traumatized. A veterinarian was recruited to monitor their hygiene and health. All sick cows were replaced and treated appropriately. During the day, the cows were allowed to graze freely in an open field. The study was performed according to relevant international animal use guidelines ^31^. This manuscript is reported in line with the ARRIVE (Animal Research: Reporting of *In Vivo* Experiments) guidelines ^32^.

## Results

### Laboratory study: Residual efficacy of VECTRON™ T500 on blocks

**Figure 1.**
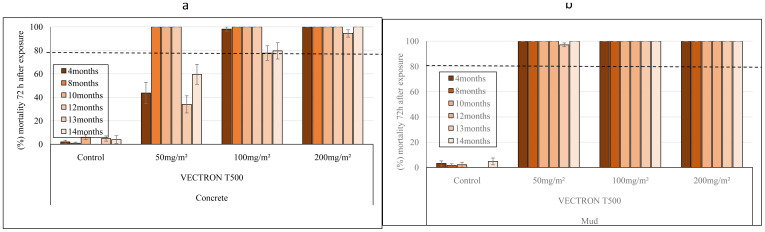
Monthly mortality of *Anopheles gambiae s.s.* Kisumu strain exposed on treated concrete ( **a**) and mud ( **b**) blocks substrates in World Health Organization (WHO) cones bioassay. Approximately 100 mosquitoes 2-5 days old were exposed for 30min to each treatment and mortality recorded 72 hours after exposure. Overall, 10 cones per dose and 10 mosquitoes per cone were used at each of tested mosquitoes at time point. Each histogram represents the mean mortality rate and error bars represent ± 95% confidence interval (CI). The dotted line represents WHO threshold.

The residual efficacy of VECTRON™ T500 was investigated at application rates of 50, 100 and 200 mg a.i./m² on concrete and mud blocks under laboratory conditions. On concrete blocks, the 50 mg a.i./m ^2^ and 100 mg a.i./m ^2^ application rates of VECTRON™ T500 resulted in 100% mortality for up to 12 months before falling below 80% during the last two months, while the 200 mg a.i./m ^2^ dose of VECTRON ^TM^ T500 gave complete mortality of susceptible *An. gambiae s.s.* Kisumu strain for up to 14 months ( Figure 1a) ^27^. VECTRON™ T500 on mud blocks showed a longer residual efficacy than on concrete blocks. Indeed, as shown in Figure 1b, all applications to mud blocks induced 100% mortality for up to 14 months after spraying with the *An. gambiae s.s.* Kisumu strain ( Figure 1b).

**Figure 2.**
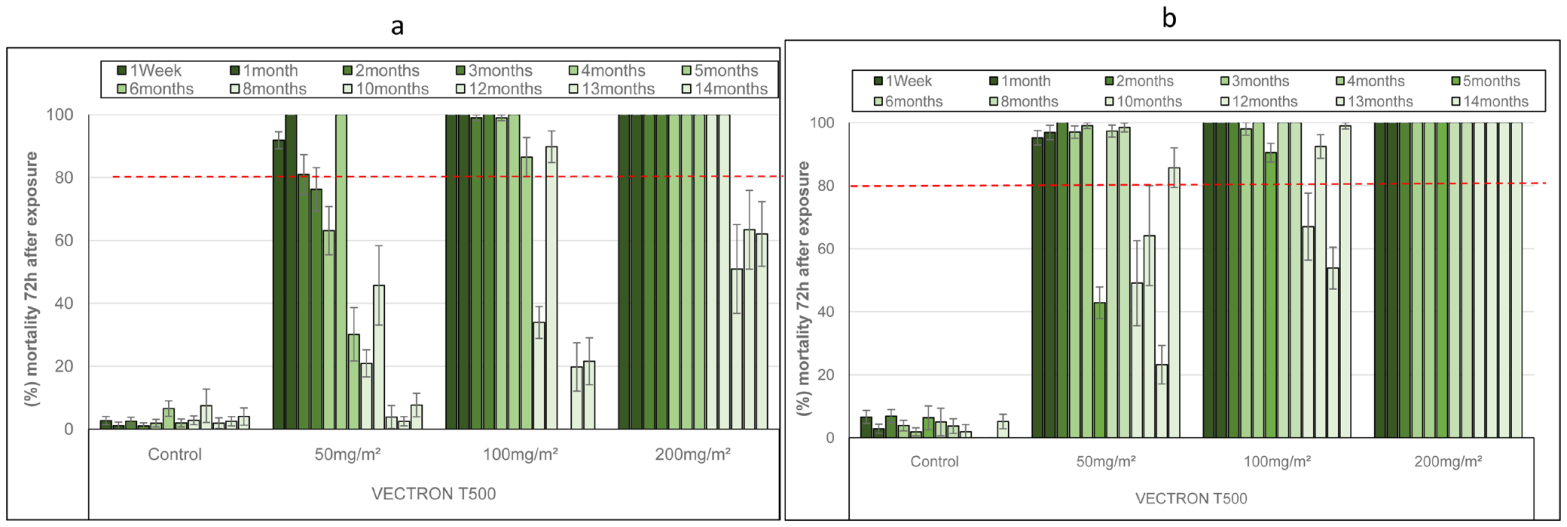
Monthly mortality of *Anopheles coluzzii* pyrethroids resistant strain exposed on treated concrete ( **a**) and mud ( **b**) blocks substrates in World Health Organization (WHO) cones bioassays. Approximately 100 mosquitoes 2-5 days old were exposed for 30min to each treatment and mortality recorded 72 hours after exposure. Overall, 10 cones per dose and 10 mosquitoes per cone were used at of tested mosquitoes at each time point. Each histogram represents the mean mortality rate and error bars represent ± 95% confidence interval (CI). The dotted line represents WHO threshold.

The residual efficacy of VECTRON™ T500 against the pyrethroid resistant VK strain of *Anopheles coluzzii* is shown in Figure 2. The results of mortality following exposure to treated concrete blocks showed dose dependent response. Indeed, for the 50 mg a.i./m ^2^ application rate, the mortality was around 80% at 5 months after block treatment before decreasing below 60% during the last six months. However, at the highest doses, the mortality rate was still high at 10 months after spraying, reaching 89.81% and 100% for the 100 mg a.i./m ^2^ and 200 mg a.i./m ^2^ doses, respectively, before decreasing below 80% during the last three months ( Figure 2a). As with the susceptible *An. gambiae s.s.* Kisumu strain, VECTRON™ T500 showed better residual efficacy on mud blocks compared with concrete blocks against the resistant VK strain of *Anopheles coluzzii*. Indeed, 100% mortality was recorded up to 14 months for the 200 mg a.i./m ^2^ application rate. The lowest application rate (50 mg a.i./m ^2^) demonstrated good residual efficacy up to 8 months after spraying with mortality up to 98.16% during that period ( Figure 2b). The 100 mg a.i./m ^2^ application rate induced mortality up to 92% 14 months after spraying, although there was some variation in mortality between months 10 and 13 ( Figure 2b).

### Experimental hut trial

***Efficacy of VECTRON ^TM^ T500 against free flying mosquitoes***

**Figure 3.**
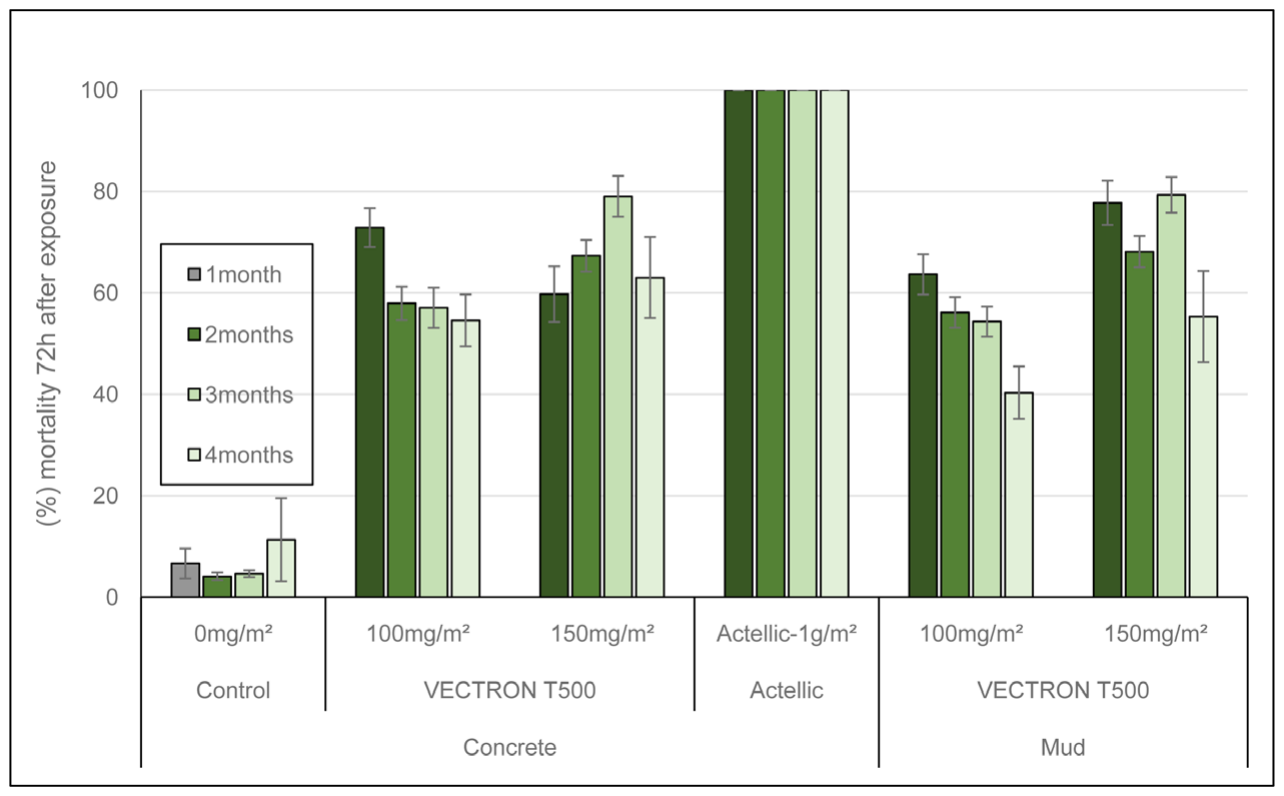
Overall mortality per month of wild free-flying pyrethroid-resistant *Anopheles gambiae* s.l. collected daily inside treated huts for 4 months evaluation. Each histogram represents monthly mean mortality rate of mosquitoes collected inside each hut during the month and error bars represent ± 95% confidence interval (CI).

Residual efficacy of VECTRON ^TM^ T500 against wild free flying pyrethroid resistant malaria vectors was investigated in an experimental hut trial at the IRSS field station in Vallée du Kou (Bama, Burkina Faso). Experimental huts simulate the conditions in domestic dwellings and are, therefore, used to assess the efficacy of indoor vector control interventions in terms of mosquito entry rates, induce early exit of vector mosquitoes, prevention of mosquito feeding and induced mosquito mortality. Cows were used in place of human volunteers for mosquito attraction in this study as mosquitoes are zoophilic and also the toxicity, and potential risk of the VECTRON ^TM^ T500 product had not been fully assessed at the time of study initiation. In addition, previous studies have shown that the local vector mosquitoes are highly attracted to blood-feed on cows. Results of the different outcome measures are presented in Figure 3 and Table 3. In total 19,552 *An. gambiae* s.l. mosquitoes were collected between August and October 2018. Mortality of free flying mosquitoes was recorded up to 72 hours after collection from huts, due to the delayed mortality effect of brofanilide, the active ingredient of VECTRON ^TM^ T500,Mortality rates of free flying *An. gambiae* s.l. indicate that the 150 mg a.i./m ^2^ dose of broflanilide was the most effective in killing mosquitoes. Indeed, this dose induced the highest overall mosquito mortality rates on concrete (70.04%) and on mud (73.22%) during the four months of mosquito collection post spraying. Statistically, the 150 mg a.i./m ^2^ dose performed significantly better (P<0.001) on mud than on concrete. During this period, mortality with the 150 mg a.i./m ^2^ dose of VECTRON™ T500 ranged from 59.74% to 79.06% on concrete walls and from 55.33% to 79.33% on mud walls ( Figure 3). Mortality of mosquitoes collected in the huts treated with 100 mg a.i./m² VECTRON™ T500 ranged from 54.57% to 72.87% on concrete walls and from 40.31% to 63.54% on mud walls with 60.40% and 55.51% as global mortality, respectively ( Figure 3 and Table 3). In contrast, 100 mg a.i./m² dose of VECTRON™ T500 performed significantly better (P<0.0001) on concrete than mud. On both substrates, there was a significant difference between150 mg a.i./m² dose and 100 mg a.i./m² in terms of mortality (P<0.0001). The positive reference product, Actellic® 300CS, induced 100% mortality during the four months of evaluation. Deterrence, blood-feeding inhibition and exophily obtained with VECTRON™ T500 treated huts compared to negative control were very low ( Table 3). The lowest of these parameters can be explained by the low action of insecticide on the mosquitoes. The Actellic® 300CS treatment showed 55% deterrence. As expected of IRS treatments, blood-feeding rates of mosquitoes were very high in all huts (>90%). There was a significant difference in blood-feeding rates between the two application rates of VECTRON™ T500 (100 mg a.i./m² and 150 mg a.i./m²) for both concrete and mud substrates (P<0.05, Table 3). The natural exophily rate in the control hut was high. Due to this natural exophily, it was not possible to determine the insecticide-induced exophily.

**Table 3.** Overall mortality 72h after collection, deterrence, blood-feeding rates and exophily induced by treatments on free flying *Anopheles gambiae* s.l. collected in treated huts during 12 consecutive weeks evaluation.

Type of wall	Concrete				Mud	
Treatments	Control	VECTRON™ T500 100 mg a.i./m²	VECTRON™ T500 150 mg a.i./m²	Actellic® 300CS mg a.i./m²	VECTRON™ T500 100 mg a.i./m²	VECTRON™ T500 150 mg a.i./m²
Total caught	3453	3546	4330	1535	3498	3190
% Deterrence	-	0	0	55.54	0	7.61
Number dead	192	2142	3033	1535	1942	2336
**Global 72h % ** **mortality**	**5.56 ^a^**	**60.40 ^b^**	**70.04 ^c^**	**100 ^d^**	**55.51 ^e^**	**73.22 ^f^**
95% CI	(4.84-6.37)	(58.78-62.00)	(68.66-71.39)	-	(53.95-57.25)	(71.76-74.73)
Blood-fed caught	3214	3277	4006	1457	3109	3038
% Blood-feeding	93.07 ^a^	92.41 ^b^	92.51 ^b^	94.91 ^c^	88.87 ^d^	95.23 ^e^
95% CI	(92.18-93.87)	(91.49-93.24)	(91.69-93.26)	(93.70-95.90)	(87.95-90.02)	(94.43-95.92)
% Blood-feeding inhibition	-	0 .71	0.60	0	4.51	0
Total exit in veranda	1139	679	1705	337	511	917
% Exophily	32.98 ^a^	19.14 ^b^	39.37 ^c^	21.95 ^d^	14.60 ^e^	28.74 ^f^
95% CI	(31.43-34.57)	(17.88-20.47)	(37.93-40.84)	(19.95-24.09)	(13.50-15.84)	(27.20-30.34)

Values bearing the same letter superscript along a row are not significantly different at the 5% level (P>0.05). CI=confidence interval.

***Residual efficacy of insecticide applied in experimental huts using cone tests***

**Figure 4.**
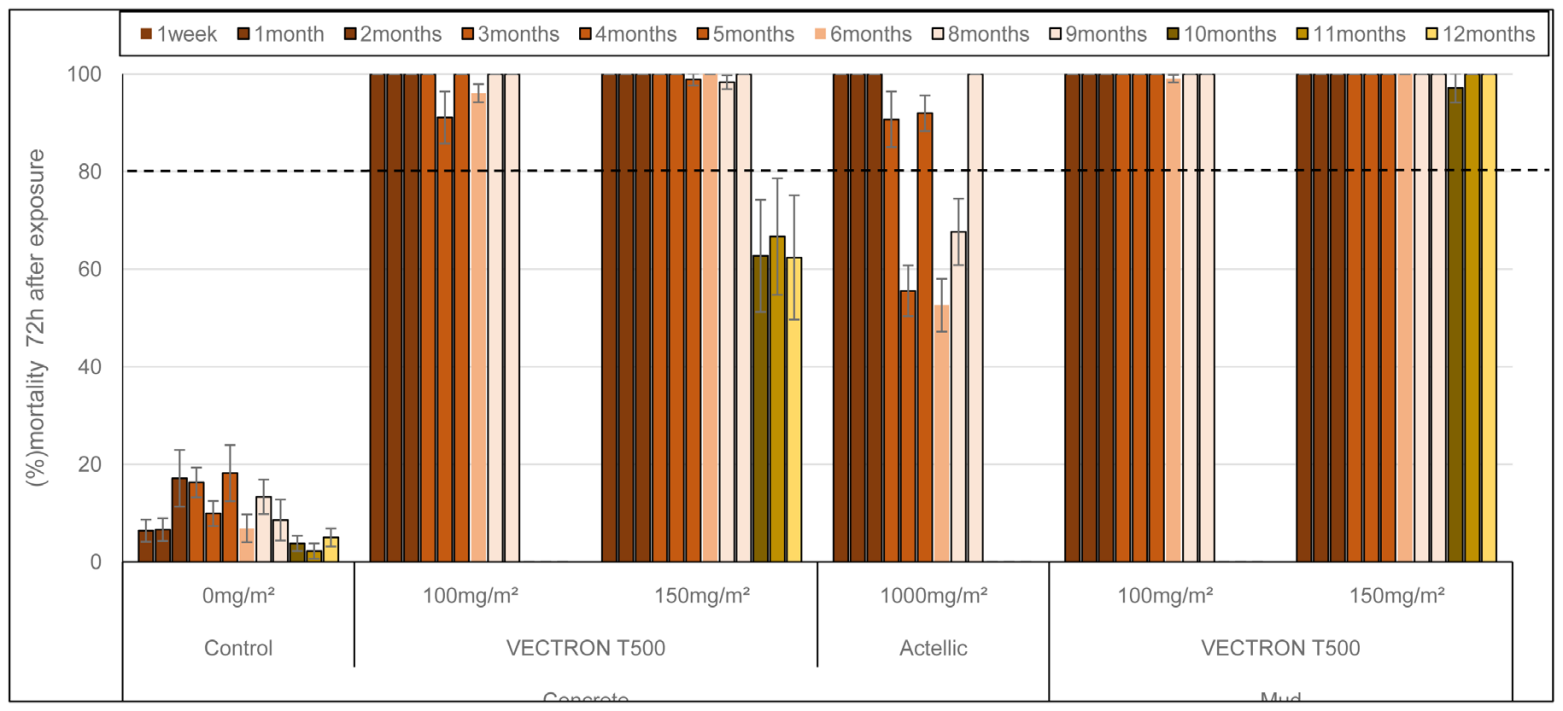
Mortality of *Anopheles gambiae* Kisumu strain exposed to treated huts surfaces in cone bioassays. Approximately 100 mosquitoes 2–5 days old were exposed for 30min to the hut walls and ceiling, and mortality recorded 72 hours after exposure. Overall, 10 cones per hut, two per side and 10 mosquitoes per cone were used at each time point. Each histogram represents the mean mortality rate of tested mosquitoes at each time point and error bars represent ± 95% confidence interval (CI). The dotted line represents World Health Organization (WHO) threshold.

**Figure 5.**
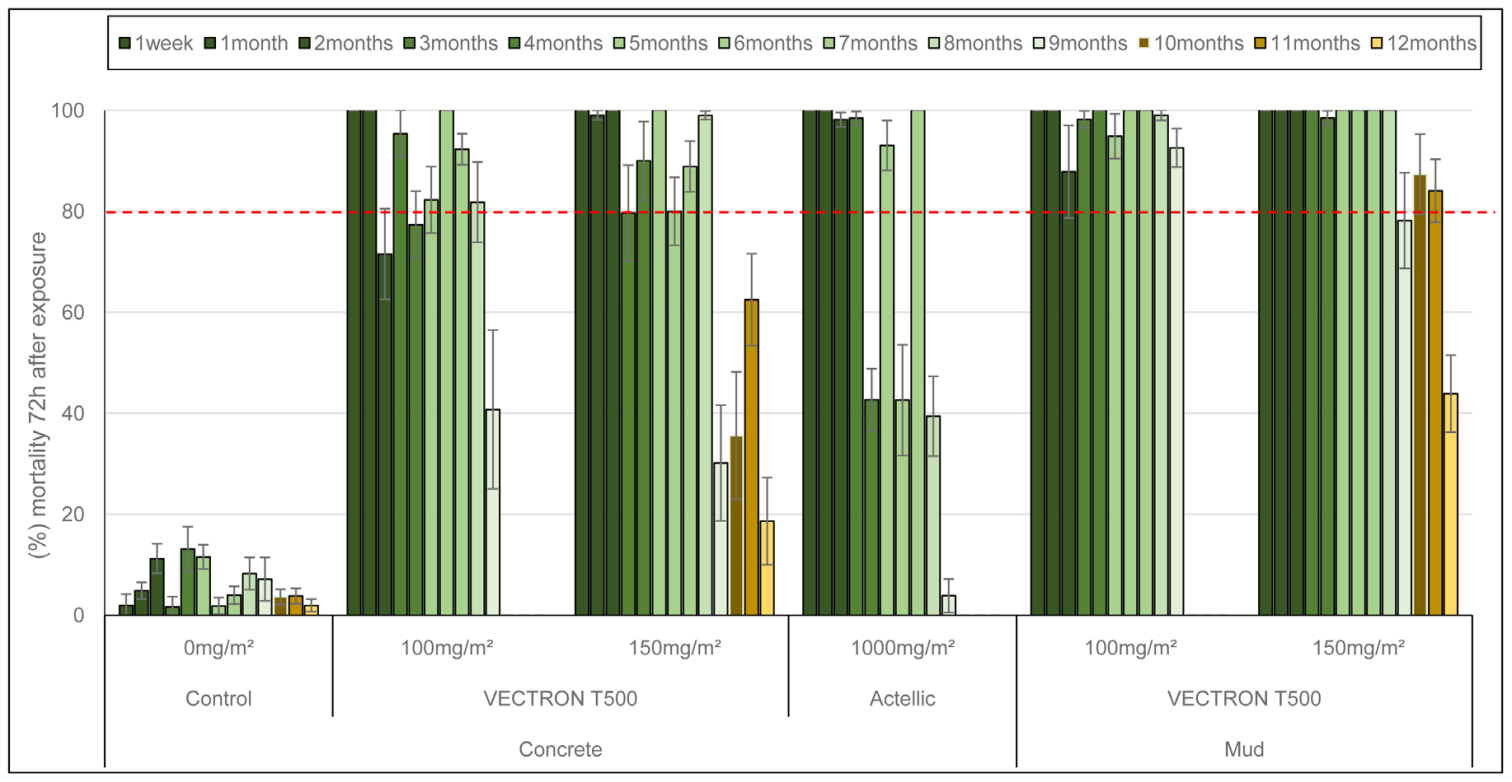
Mortality of *Anopheles coluzzii* pyrethroids resistant strain exposed to treated huts surfaces in cone bioassays. Approximately 100 mosquitoes 2–5 days old were exposed for 30min contact to the treated hut walls and ceiling, and mortality recorded 72 hours after exposure. Overall, 10 cones per hut, two per side and 10 mosquitoes per cone were used at each time point. Each histogram represents the mean mortality rate of tested mosquitoes at each time point and error bars represent ± 95% confidence interval (CI). The dotted line represents World Health Organization (WHO) threshold.

Cone bioassays were performed monthly in experimental huts up to 9 months for the 100 mg a.i./m ^2^ application rate of VECTRON™ T500 and for the Actellic® 300CS treatment. The 150 mg a.i. /m ^2^ application rate of VECTRON™ T500 was evaluated up to 12 months after spraying, to assess the residual efficacy on the different hut wall substrates (mud and concrete). Unfed adult females (3–5 days old) of the susceptible *An. gambiae* Kisumu and resistant *An. coluzzii* (reared from****** larvae collected at the experimental field site) were used. The residual efficacy in cone bioassays with the susceptible Kisumu strain is presented in Figure 4. The 100 mg/m ^2^ and 150 mg/m ^2^ doses of VECTRON™ T500 induced 100% mortality on both concrete and mud walls up to 9 months after spraying. The three extended monthly test performed with 150 mg/m² showed better efficacy up to 12 months on mud walls (100%) than concrete walls (<80%). The Actellic® 300CS reference product applied to concrete showed variable mortality from 4 months post-treatment onwards, and mortality at 6 and 7 months was below 80%. Residual efficacy against the pyrethroid resistant VK strain *An. coluzzii* is shown in Figure 5. The 100 mg a.i./m² and 150 mg a.i./m ^2^ doses of VECTRON™ T500 performed better on mud walls (mortality over 80%) 9 months after spraying than on concrete walls (mortality below 80%) at the same time. The Actellic® 300CS showed variable residual efficacy from 4 months post-treatment onwards, but mortality was below 80% at 8 and 9 months.

### Insecticide application quality

The results of the HPLC analysis of filter papers treated during the application of treatments to experimental hut walls are shown in Table 4. The percentage difference between the target dose and the actual dose sprayed onto filter papers was within the range of ±50% of the target doses for all treatments confirming that the spraying met WHO statement for spray quality ^26^.

**Table 4.** Insecticide application quality results obtained by filter papers treated during the huts spraying analysis.

Walls	Concrete			Mud	
Treatments	VECTRON™ T500	VECTRON™ T500	Actellic 300CS	VECTRON™ T500	VECTRON™ T500
**Target doses (mg/m²)**	100	150	1000	100	150
**Filter paper doses (mg/m²)**	109.17	188.84	800.55	121.14	150.90
**Deviation from target doses (%)**	9.17	25.89	-19.94	21.14	0.60

### WHO susceptibility assays

To determine the prevalence of phenotypic resistance, larvae were collected from breeding sites near to the experimental hut station and reared in the insectary to adults 2–5 days old. WHO susceptibility bioassays were performed using insecticide impregnated papers obtained from the Vector Control Research Unit (VCRU) WHO Collaborating Centre, University Sains Malaysia, Penang, Malaysia in Malaysia. The insecticide susceptible Kisumu strain of *An. gambiae s.s.* was also tested for data quality control purposes. The results summarized below ( Table 5) showed full susceptibility (100% mortality) of the Kisumu strain and a very high level of resistance to pyrethroids (<2% mortality) in the field population of *An. gambiae* s.l. The increase in mortality (39%) with deltamethrin following exposure to the cytochrome P450 synergist piperonyl butoxide (PBO), indicates the role of a P450 metabolic mechanism of pyrethroid resistance in this population. Resistance was also observed in the field population to the carbamate bendiocarb (84%), but it was fully susceptible to the organophosphorus insecticide pirimiphos-methyl.

**Table 5.** WHO susceptibility assay results, Knock down and mortality of mosquitoes tested in World Health Organization (WHO) tube using impregnated papers to evaluate phenotypic resistance.

treatments	Susceptible *An. gambiae* Kisumu strain			*An. gambiae s.l.* from field larval collections		
	number tested	% (KD)	% mortality 24h	number tested	% (KD)	% mortality 24h
Control PY	54	0	4	54	0	0
Permethrin_0,75%	103	100	100	100	0	0
Alpha-cypermethrin_0,05%	100	100	100	102	0	0
Deltamethrin_0,05%	102	100	100	105	0.93	1.87
Control/OP	54	0	0	52	0	1.96
Pirimiphos_methyl_0.25%	105	100	100	101	12	99
Bendiocarb_0,1%	101	100	100	99	93088	84.69
Control/PBO (4%)	-	-	-	53	0	0
PBO (4%) + Deltamethrin_0,05%	-	-	-	99	52.53	39.39

%: Percentage, KD: Knock down, PY: Pyrethroids, OP: Organophosphate, PBO: piperonyl butoxide.

### Species identification and kdr genotyping

Mosquitoes sampled from treated huts (610 individual mosquitoes) were used in PCR tests to determine species and to detect *kdr* resistance mutations. Of these mosquitoes, 8 mosquitoes did not amplify. A high proportion of the mosquitoes collected from huts were *An. coluzzii* (98%), and these had a high frequency of the *kdr* (L1014F) mutation (0.65). The proportion of heterozygote, homozygote resistance and homozygote susceptible was 42.6%, 44.5% and 12.8% respectively ( Table 6). The *kdr* mutations confer cross-resistance between pyrethroids and DDT in these mosquitoes.

**Table 6.** Species identification and kdr genotyping by polymerase chain reaction (PCR).

Species	Number	Genotypes of the kdr-w			f(L1014F)
		1014L 1014L	1014L 1014LF	1014F 1014LF	
*An. coluzzii*	591(98%)	76(12.8%)	252(42.6%)	263(44.5%)	0.65
*An. gambiae*	10(1.6%)	0	0	10(100%)	1
*An. arabiensis*	1(0.1%)	0	0	1(100%)	1
total	602	76(12.6%)	252(41.8)	274(45.5)	0.66

f(1014F) : frequency of the 1014F resistant *kdr* allele.

## Discussion

Control of malaria vectors is dominated by use of insecticides on LLINs and applied by IRS. Unfortunately, the major malaria vector species have become resistant to many of the classes of insecticides currently recommended by WHO for use in public health ^11^. Managing insecticide resistance is a major challenge for malaria control and elimination ^33^ and implementation of insecticide resistance management strategies is a key method for the continued control of malaria. Such strategies require new insecticides, with modes of action effective against resistant strains of mosquito ^34, 35^.

To address the urgent need for new insecticides with novel modes of action to control malaria vectors, we investigated the bioefficacy of broflanilide in a wettable powder formulation, VECTRON™ T500, for use in IRS, through the conduct of laboratory (Phase I) and experimental hut (Phase II) studies. Insecticide susceptibility assays showed high resistance of *An. gambiae* s.l. to all pyrethroids tested (deltamethrin, permethrin and alphacypermethrin) and moderate resistance to carbamate (bendiocarb) in the malaria vector population in Vallée du Kou, Burkina Faso. The molecular diagnostic (PCR) testing detected a high frequency of the kdr (L1014F) mutation in the mosquito population. Recent studies of this population have shown a high resistance to the three main classes of insecticides (DDT, carbamate and pyrethroids) used in vector control throughout the country ^10^. Pre-exposure to the synergist PBO in bioassays with deltamethrin increased mortality, suggesting the presence of a cytochrome P450-based mechanism of resistance in this mosquito population. However, pre-exposure to PBO did not fully restore the susceptibility of the mosquitoes to deltamethrin, which indicates the presence of other resistance mechanisms ^8, 10^.

The results of the laboratory and experimental hut trials clearly demonstrate the ability of brofanilide insecticide to give high levels of mortality (>80%) in pyrethroid resistant mosquitoes up to 6 months post-spraying. The lowest dose tested in the experimental hut study, 100 mg a.i./m ^2^, gave more than 80% mortlity 6 months after application to mud or concrete in laboratory cone bioassays and *in situ* cone tests carried out in the experimental huts. Similar results were reported in other studies performed in Benin and Tanzania with *An. gambiae* s.l. and *An. Arabiensis,* respectively ^36, 37^. The residual activity through *in situ* cone bioassays on treated experimental huts walls with susceptible and pyrethroid-resistant vector mosquito strains indicates that VECTRON™ T500 performed as well as Actellic® 300CS, a WHO listed IRS product, during 4 to 9 months post-spraying. This demonstrates the potential of VECTRON™ T500 to provide prolonged vector control in many malaria-endemic African villages where the interiors of houses are largely plastered with mud only. High levels of mortality were seen with free flying mosquitoes entering huts treated with VECTRON™ T500 or Actellic® 300CS during the four months of evaluation. Residual efficacy of Actellic® 300CS in the present study is similar to the findings of a previous study ^38^. Both 100 mg a.i./m ^2^ and 150 mg a.i./m ^2^ applications of VECTRON ^TM^ T500 gave extended residual efficacy in experimental huts, residual efficacy being longer on mud substrate than on concrete. With its novel mode of action and efficacy for IRS against pyrethroid-resistant malaria vectors, VECTRON™ T500 shows potential for use with other IRS insecticide formulations in an IRS rotation strategy to help manage insecticide resistance and extend the effective lives of the insecticides used in IRS.

## Conclusion

The laboratory and experimental hut trials reported here have demonstrated the extended residual efficacy of VECTRON™ T500, a wettable powder formulation of broflanilide, against both susceptible and pyrethroid-resistant mosquito strains for 6 months or more, post-spraying onto mud and concrete substrates. The present study has defined the dose for VECTRON™ T500 to be used in Community trials i.e. 100 mg a.i./m².

## Data availability

### Underling data

Zenodo: koamabayili/VECTRON: Laboratory and experimental hut trial evaluation of VECTRON™ T500 for indoor residual spraying (IRS) against insecticide resistant malaria vectors in Burkina Faso. https://doi.org/10.5281/zenodo.6469836 ^27^.

This project contains the following underlying data:

- file 1-laboratory cone test raw data.xlsx
- file 2-free flying raw data.xlsx
- file 3-residual efficacy inside huts raw data.xlsx
- file 4-filter papers analysis raw data.xlsx
- file 5-PCR raw data.xlsx
- file 9-PCR revelation for especies.pdf
- file 10-PCR revelation for kdr.pdf

### Extended data

Zenodo: koamabayili/VECTRON: Laboratory and experimental hut trial evaluation of VECTRON™ T500 for indoor residual spraying (IRS) against insecticide resistant malaria vectors in Burkina Faso. https://doi.org/10.5281/zenodo.6469836 ^27^.

This project contains the following extended data:

- file 6-script of laboratory data.R
- file 7-script of free flying data.R
- file 8-script of residual efficacy data.R
- file 11-PCR reagents and equipments details.docx

### Reporting guidelines

Zenodo: ARRIVE checklist for ‘Laboratory and experimental hut trial evaluation of VECTRON™ T500 for indoor residual spraying (IRS) against insecticide resistant malaria vectors in Burkina Faso’.

https://doi.org/10.5281/zenodo.6469817 ^32^.

Data are available under the terms of the Creative Commons Zero "No rights reserved" data waiver (CC0 1.0 Public domain dedication).
