# Supplementary material for: Laboratory and experimental hut trial evaluation of VECTRON ™ T500 for indoor residual spraying (IRS) against insecticide resistant malaria vectors in Burkina Faso
Source: Gates Open Res. 2022 Jul 25;6:57. Originally published 2022 Apr 25. [Version 2] doi: 10.12688/gatesopenres.13578.2 (PMC9326088; doi:10.12688/gatesopenres.13578.2)
Supplement: Peer review history

# Peer review: Reviewer response for version 2

**DOI:** 10.21956/gatesopenres.14961.r32300

**Published:** 2022-08-11

**Authors:** Rosemary S. Lees, George Parsons, Giorgio Praulins, Katherine Gleave

**Affiliations:** 1 Department of Vector Biology, Liverpool School of Tropical Medicine, Liverpool, UK

I am satisfied and I confirm that the author has addressed my comments adequately. I approve of the manuscript,

Is the work clearly and accurately presented and does it cite the current literature?

Yes

If applicable, is the statistical analysis and its interpretation appropriate?

I cannot comment. A qualified statistician is required.

Are all the source data underlying the results available to ensure full reproducibility?

Yes

Is the study design appropriate and is the work technically sound?

Yes

Are the conclusions drawn adequately supported by the results?

Yes

Are sufficient details of methods and analysis provided to allow replication by others?

Partly

Reviewer Expertise:

Medical entomology, development and evaluation of vector control tools, insecticide resistance

We confirm that we have read this submission and believe that we have an appropriate level of expertise to confirm that it is of an acceptable scientific standard.

# Peer review: Reviewer response for version 2

**DOI:** 10.21956/gatesopenres.14961.r32298

**Published:** 2022-07-25

**Authors:** Emmanuel Mbuba

**Affiliations:** 1 Vector Control Product Testing Unit, Environmental Health and Ecological Science Department, Ifakara Health Institute, Bagamoyo, Tanzania

I am satisfied and I confirm that the author has addressed my comments adequately. I approve of the manuscript

Is the work clearly and accurately presented and does it cite the current literature?

Yes

If applicable, is the statistical analysis and its interpretation appropriate?

Yes

Are all the source data underlying the results available to ensure full reproducibility?

Yes

Is the study design appropriate and is the work technically sound?

Partly

Are the conclusions drawn adequately supported by the results?

Yes

Are sufficient details of methods and analysis provided to allow replication by others?

Yes

Reviewer Expertise:

NA

I confirm that I have read this submission and believe that I have an appropriate level of expertise to confirm that it is of an acceptable scientific standard.

# Response

**Published:** 2022-07-25

**Authors:** koama bayili

**Affiliations:** Institut de recherche en sciences de la santé, Burkina Faso

Dear Emmanuel Mbuba

 Thank you very much for your time and your improved comments and corrections that ameliorated manuscript quality.

# Peer review: Reviewer response for version 1

**DOI:** 10.21956/gatesopenres.14848.r32087

**Published:** 2022-06-16

**Authors:** Rosemary S. Lees, Katherine Gleave, Giorgio Praulins, George Parsons

**Affiliations:** 1 Department of Vector Biology, Liverpool School of Tropical Medicine, Liverpool, UK

Koama Bayili *et al.* have conducted an important piece of evaluation work for the VECTRON T500 IRS product, which follows on in a logical fashion from previously published laboratory evaluation of the active ingredient Tenebenal (Lees *et al* 2020) ^1^. Standard laboratory and semi-field methods have been applied by an experienced team to demonstrate the residual activity of the IRS formulation applied to mud and concrete blocks in controlled laboratory conditions, and to the walls of mud and concrete experimental huts, against laboratory colonies and free-flying wild mosquitoes, respectively. Experiments were conducted according to the WHO guidelines, with a good level of quality control and additional checks, for example, the information about training, HPLC analysis, and genotyping as well as phenotyping of free flying mosquitoes.

 There are some small points of clarification and suggestions for improvement below. The main limitation of the manuscript is that some of the methodological detail is not very well described, and so the experimental design is confusing in some places. Although the study type will be familiar to many readers, to those who are less familiar with this form of testing it would be hard to replicate the experiments.

 General:

- There is some confusion over how the product under evaluation is referred to - it is sometimes called VECTRON T500, sometimes just VECTRON, and in Figures 4 and 5, for example, as Tenebenal 50WP. It seems like the same product was used throughout, but it would be helpful to either explain the different names in the Introduction or ideally be consistent in the naming.
- There are references to two versions of the WHO guidelines for evaluation of IRS products, which do not differ in the methods they present but it would be better to cite them consistently.
- There is some repetition between the Methods section and Introduction.
- There are some typos and misspellings, for example, the use of capital letters for species names and reference to ‘resistance mosquitoes’ rather than ‘resistant’.

Introduction:

- It would be useful to have a sentence to explain the need for the 72h observation of mortality, which is not standard but required for this chemistry which is slower acting than pyrethroids. This could be included in the section of the Introduction which describes Tenebenal, and which would be usefully expanded to introduce the new chemistry to readers who are not familiar with it.
- The Introduction uses WHOPES terminology to refer to Phase I and Phase II testing, which could be updated to reflect current WHO PQT/VCP terminology.

Methods:

- I would like to see some more detail in the Methods section, for example, details on the batch number and quality control of the product being used in this study, and how the Potter Tower was calibrated. If HPLC or other analysis was done to confirm the rate of application by the Potter Tower I would like to see this data. In particular, I found it difficult to understand the experimental hut trial procedure – how many rounds of 6 nights of testing were completed over the 12 weeks of testing, how the huts and cows were rotated, etc. A schematic or diagram of some sort would be helpful for readers not familiar with this type of study.
- It would be nice to see a justification for why the concentrations of IRS changed between the laboratory and experimental hut phase of testing.
- It is helpful that the authors shared their R scripts, though they should also be cited appropriately in the text. It would also be helpful if they shared the code for the mixed effects model, to allow results to be reproduced.

Results/Discussion:

- The very low blood feeding inhibition, deterrence, and exophily in the experimental hut experiment is interesting, alongside good levels of mortality. I would like to see the authors discuss the implications of this for the expected performance of the product against pyrethroid resistant populations of mosquitoes.

Is the work clearly and accurately presented and does it cite the current literature?

Yes

If applicable, is the statistical analysis and its interpretation appropriate?

I cannot comment. A qualified statistician is required.

Are all the source data underlying the results available to ensure full reproducibility?

Yes

Is the study design appropriate and is the work technically sound?

Yes

Are the conclusions drawn adequately supported by the results?

Yes

Are sufficient details of methods and analysis provided to allow replication by others?

Partly

Reviewer Expertise:

Medical entomology, development and evaluation of vector control tools, insecticide resistance

We confirm that we have read this submission and believe that we have an appropriate level of expertise to confirm that it is of an acceptable scientific standard.

## References

[1] : Tenebenal: a meta-diamide with potential for use as a novel mode of action insecticide for public health. *Malar J* .2020;19(1) : 10.1186/s12936-020-03466-4 398 10.1186/s12936-020-03466-4 33168015PMC7654575

# Response

**Published:** 2022-06-22

**Authors:** koama bayili

**Affiliations:** Institut de recherche en sciences de la santé, Burkina Faso

**Dear Rosemary S. Lees *et al.***

**We thank you and your colleagues for your constructive comments and suggestions to improve the manuscript. The manuscript will be revised accordingly. All changes or additions in the text will be done in the revised version before submitting it. All the changes will be in blue in the revised version.**

**I tried to respond to your below comments:**

 There is some confusion over how the product under evaluation is referred to - it is sometimes called VECTRON T500, sometimes just VECTRON, and in Figures 4 and 5, for example, as Tenebenal 50WP. It seems like the same product was used throughout, but it would be helpful to either explain the different names in the Introduction or ideally be consistent in the naming.

**Response: I corrected the word and the right name is VECTRON T500**

 There are references to two versions of the WHO guidelines for evaluation of IRS products, which do not differ in the methods they present but it would be better to cite them consistently

 .

**Response: Yes** 

 There is some repetition between the Methods section and Introduction.

**Response: Yes, we just want some time to remind you about some right information.**

 There are some typos and misspellings, for example, the use of capital letters for species names and reference to ‘resistance mosquitoes’ rather than ‘resistant’.

**Response: Yes, corrected**

 Introduction:

- It would be useful to have a sentence to explain the need for the 72h observation of mortality, which is not standard but required for this chemistry which is slower acting than pyrethroids. This could be included in the section of the Introduction which describes Tenebenal, and which would be usefully expanded to introduce the new chemistry to readers who are not familiar with it.
**Response: Yes included**

 

- The Introduction uses WHOPES terminology to refer to Phase I and Phase II testing, which could be updated to reflect current WHO PQT/VCP terminology. 
**Response: Yes, corrected**

Methods:

- I would like to see some more detail in the Methods section, for example, details on the batch number and quality control of the product being used in this study, and how the Potter Tower was calibrated. If HPLC or other analysis was done to confirm the rate of application by the Potter Tower I would like to see this data. In particular, I found it difficult to understand the experimental hut trial procedure – how many rounds of 6 nights of testing were completed over the 12 weeks of testing, how the huts and cows were rotated, etc. A schematic or diagram of some sort would be helpful for readers not familiar with this type of study.
**Response: The batch number of products is included. Unfortunately, we missed the potter Tower calibration results that would be filter papers analysis. We did 12 rounds with 6 nights each. The huts were not rotated only the cows were rotated as in the example below:**

**Hut**

**Number    1    2      3        4      5       6**

**Day 1    C1    C2    C3    C4    C5    C6**

**Day 2    C2    C3    C4    C5    C6    C1**

**Day 3    C3    C4    C5    C6    C1    C2**

**Day 4    C4    C5    C6    C1    C2    C3**

**Day 5    C5    C6    C1    C2    C3    C4**

**Day 6    C6    C1    C2    C3    C4    C5**

**C1 to C6 refers to cows**

  

- It would be nice to see a justification for why the concentrations of IRS changed between the laboratory and experimental hut phase of testing.
**Response: As in the lab 100mg/m² was performed as 200mg/m² and the product production cost is very expensive, we judged that it will be good to reduce the concentration in the field.**
- It is helpful that the authors shared their R scripts, though they should also be cited appropriately in the text. It would also be helpful if they shared the code for the mixed-effects model, to allow results to be reproduced.
**Response: Please see in the data availability section R scripts are in files 6, 7, and 8.**

Results/Discussion:

- The very low blood feeding inhibition, deterrence, and exophily in the experimental hut experiment is interesting, alongside good levels of mortality. I would like to see the authors discuss the implications of this for the expected performance of the product against pyrethroid resistant populations of mosquitoes.
**Response: I tried to explain it in the results section by the lowest action of insecticide on the mosquitoes.**

**Thank you very much again for your time in reviewing this manuscript**

# Peer review: Reviewer response for version 1

**DOI:** 10.21956/gatesopenres.14848.r32086

**Published:** 2022-06-07

**Authors:** Emmanuel Mbuba

**Affiliations:** 1 Vector Control Product Testing Unit, Environmental Health and Ecological Science Department, Ifakara Health Institute, Bagamoyo, Tanzania

**REVIEWER SUMMARY**

 General comment on the paper: The authors provide background to why it is important to develop and evaluate new indoor residual spray formulations following the increase and wide spread of mosquito resistance against currently used vector interventions mainly long-lasting insecticidal nets (LLINs) and indoor residual spray (IRS). The authors introduce VECRTONTM T500, a novel insecticide formulation that may have potential as a new IRS vector control product.

 The objectives of the study are clearly stated as 1) to determine the dose required for the application of  VECRTONTM T500  and its residual efficacy in the lab and in the wild mosquito population. The study and the experiment were conducted in Latin-square design. Although the study was a Latin-square, it is not stated why the positive control Actellic 300CS was not tested in mud which would provide a 7 x 7 treatment arms.

  Here are minor comments that I suggest the author correct/clarify before indexing:

  

1. Currently, WHO PQT does not use Phase I or Phase studies. Instated laboratory study & Experimental hut should be used.  I suggest correcting this throughout the manuscript.
2. In the lab study, the author used  50, 100, and 200 mg/m2  but in the experimental hut study, the author decided to use 150 mg/m2. No statement about this decision
3. On page 5, the author mentions that cows were used because wild mosquitoes are zoophilic. On page 7, the author mentions that cow was used because the safety profile of VECRTONTM T500 is not established. This is confusing. In addition, it raises serious safety concerns and really suggests the author needs to clarify or correct this.
4. There is inconsistency in figure keys, labels, and legends.
5. On page 9, the authors included numbers which I failed to find in the table of results. I suggest clarifying which figure or table they are linked to. Or if it is not shown, it should be stated.
6. In the discussion section, the author is reintroducing the subject using several sentences, which I think are not necessary and makes the discussion unnecessarily long. 
7. All my comments are in the manuscript I reviewed. I suggest the author have a look and address them whenever necessary. ( Bayili *et al* feedback.pdf) 
8. It is odd that the author did not include a section for study limitations.  I noted several limitations. 1) It would be good to use WHO papers with 5x discriminating concentration as this gives important info on the resistance intensity. The author did not do a second 7x7 latin square to look at effects up to 8 months post spray. This is important when the Vectron looks better than Actellic in the cone test.

Is the work clearly and accurately presented and does it cite the current literature?

Yes

If applicable, is the statistical analysis and its interpretation appropriate?

Yes

Are all the source data underlying the results available to ensure full reproducibility?

Yes

Is the study design appropriate and is the work technically sound?

Partly

Are the conclusions drawn adequately supported by the results?

Yes

Are sufficient details of methods and analysis provided to allow replication by others?

Yes

Reviewer Expertise:

NA

I confirm that I have read this submission and believe that I have an appropriate level of expertise to confirm that it is of an acceptable scientific standard, however I have significant reservations, as outlined above.

# Response

**Published:** 2022-06-22

**Authors:** koama bayili

**Affiliations:** Institut de recherche en sciences de la santé, Burkina Faso

**Dear** **Emmanuel Mbuba**

**We thank you for your constructive comments and suggestions to improve the manuscript. The manuscript will be revised accordingly.**

 As your comments and suggestion are below, I tried to respond to them in the revised manuscript. Please see my comments in bold.

1. Currently, WHO PQT does not use Phase I or Phase studies. Instated laboratory study & Experimental hut should be used.  I suggest correcting this throughout the manuscript.
**Response:  All corrections are done accordingly **
2. In the lab study, the author used  50, 100, and 200 mg/m2  but in the experimental hut study, the author decided to use 150 mg/m2. No statement about this decision
**Response: You are right. In the lab, 100mg/m² was performed as 200mg/m² and we don't want to use much product due to its production cost, which is why we reduced the dose.**
3. On page 5, the author mentions that cows were used because wild mosquitoes are zoophilic. On page 7, the author mentions that cow was used because the safety profile of VECRTONTM T500 is not established. This is confusing. In addition, it raises serious safety concerns and really suggests the author needs to clarify or correct this.
**Response: I tried to correct and clarify this in the manuscript**
4. There is inconsistency in figure keys, labels, and legends.
**Response: All corrections are done accordingly **
5. On page 9, the authors included numbers which I failed to find in the table of results. I suggest clarifying which figure or table they are linked to. Or if it is not shown, it should be stated.
**Response: All corrections are done accordingly reference was done to Figure 3 and Table 3.**
6. In the discussion section, the author is reintroducing the subject using several sentences, which I think are not necessary and makes the discussion unnecessarily long. 
**Response: All corrections are done and discussion is reduced**
7. All my comments are in the manuscript I reviewed. I suggest the author have a look and address them whenever necessary. ( Bayili *et al* feedback.pdf) 
**Response:** **Yes I got them and took them in count on the revised manuscript**
8. It is odd that the author did not include a section for study limitations. I noted several limitations. 1) It would be good to use WHO papers with 5x discriminating concentration as this gives important info on the resistance intensity. The author did not do a second 7x7 latin square to look at effects up to 8 months post spray. This is important when the Vectron looks better than Actellic in the cone test. 
**Response:** **Yes these are the limitation of our study and we will consider these suggestions in other studies that will be run in the same area.**

**Thank you very much again for your time in reviewing this manuscript.**

# Peer review: Reviewer response for version 1

**DOI:** 10.21956/gatesopenres.14848.r32090

**Published:** 2022-06-07

**Authors:** Johnson J. Matowo

**Affiliations:** 1 Departmentof Parasitology and Entomology, Faculty of Medicine, Kilimanjaro Christian Medical University College, Moshi, Tanzania

Reviewer's report on the manuscript titled **Laboratory and experimental hut trial evaluation of VECTRON ^™^ T500 for indoor residual spraying (IRS) against insecticide resistant malaria vectors in Burkina Faso**

 Reviewer: **Dr. Johnson Matowo (PhD)**

 The manuscript by Bayili *et al.* investigated the optimum effective dose and efficacy of VECTRON™ T500  against susceptible and resistant strains of Anopheles in Burkina Faso

**Title:**

 Seems to be okay

**Abstract:**

 World Health Organization recommended insecticides for vector control are limited in number

 To be rephrased as follows:

 The World Health Organization (WHO) recommended insecticides for vector control are limited in number

**Background**

 Okay

**Methods**

1. The title Chemical residue analysis is to be changed to Insecticide application quality
2. Supplementary tests: To be changed from Phenotypical and genotypical resistance to Polymerase Chain Reactions (PCR) and WHO resistance assay
3. **Page 6-7**
  The following text
*Phenotypical resistance was evaluated by using the WHO tube test method30. Larvae were collected from the field site and reared in the insectary to adults 2 to 5 days old. Papers impregnated with pyrethroids, carbamate, organophosphate insecticides and the synergist piperonyl butoxide (PBO) from the WHO laboratory in Malaysia were used. *
  Could be rephrased as follows:
  Phenotypic resistance was evaluated using 2 to 5 -day old adult female mosquitoes according to the standard WHO susceptibility test method ^30^. The insecticide and PBO  treated papers (impregnated papers) were obtained from WHO laboratory in Malaysia.
4. **Page 7**
  The following sentence
*In the experimental hut trial, the free flying mosquito data such as the number of mosquitoes that entered, exited, or were dead inside the hut…….;* seems to be incomplete. This should be addressed.
5. **Page 5:** The local *An. coluzzii* population is relatively zoophilic. Please provide a citation for this

**Results**

1. The title Phenotypical and genotypical resistance is to be deleted. Instead, the text could be placed under two headings
  WHO susceptibility assays
  Phenotypic resistance data (%mortality and %knockdown data)
2. Species identification and kdr genotyping
  Species composition and *kdr *mutations data
3. The first paragraph of each subsection of the Results section is part of methodology section. This is to be shifted to the methodology section.
4. **Figures**
  Figure legends
  The second sentence for figure legends figures 1,2,4, and 5 is part of the methodology section and should be deleted. For example *figure 1: Approximately 100 mosquitoes 2-5 days old were exposed for 30min to each treatment and mortality was recorded 72 hours after exposure. Overall, 10 cones per dose and 10 mosquitoes per cone were used at each of tested mosquitoes at time point*
5. **Tables**
  Headings for tables 4,5, and 6 are to be rephrased as follows

- Table 4: HPLC analysis or Insecticide application quality
- Table 5: WHO susceptibility assay
- Table 6: Species identification and kdr genotyping
  The following should appear as footnotes, below the tables, not on the heading
- Table 3: Values bearing the same letter superscript along a row are not significantly different at the 5% level (P>0.05). CI=confidence interval
- Table 5:  %: Percentage, KD: Knock down, PY: Pyrethroids, OP: Organophosphate, PBO: piperonyl butoxide
- Table 6: f(1014F): frequency of the 1014F resistant kdr allele
  The format of ALL tables could be formatted. Instead of highlights, the tables to have few horizontal lines (column headings and base of tables) with no vertical lines
  For table 5, two cells for strains and treatments to be merged and the word strains to be deleted, the cell to be for treatments.

**Discussion**

 The authors are not focused. There is unnecessary background information on background and methodology.

1. The first paragraph could be followed by the following paragraph
*To address the urgent need for new insecticides with novel modes of action to control malaria vectors, we investigated the bioefficacy of broflanilide in a wettable powder formulation, VECTRON™ T500, for use in IRS, through the conduct of laboratory (Phase I) and experimental hut (Phase II) studies. Insecticide susceptibility assays showed high resistance of An. gambiae s.l. to all pyrethroids tested (deltamethrin, permethrin and alphacypermethrin) and moderate resistance to carbamate (bendiocarb) in the malaria vector population in Vallée du Kou, Burkina Faso. The molecular diagnostic (PCR) testing detected a high frequency of the kdr (L1014F) mutation in the mosquito population. Recent studies of this population have shown a high resistance to the three main classes of insecticides (DDT, carbamate and pyrethroids) used in vector control throughout the country10. Pre-exposure to the synergist PBO in bioassays with deltamethrin increased mortality, suggesting the presence of a cytochrome P450-based mechanism of resistance in this mosquito population. However, pre-exposure to PBO did not fully restore the susceptibility of the mosquitoes to deltamethrin, which indicates the presence of other resistance mechanisms ^8,10^*
2. Then the following text
*Broflanilide, discovered by Mitsui Chemicals Agro, Inc., has an unique chemical structure characterized as a meta-diamide and shows strong activity against various pests ^15^. This insecticide, with a new mode of action for malaria vector control, shows no cross-resistance to existing pyrethroid resistance mechanisms ^34^. This is an important consideration for future use in IRS product rotations for insecticide resistance management purposes. VECTRON™ T500 was tested as an IRS against pyrethroid susceptible and pyrethroid-resistant strains of malaria vectors following application to mud and concrete, the two principal substrates used in Bama village, Vallée du Kou, inside the houses. Residual efficacy was assessed via cone bioassays and by assessing mortality of free flying mosquitoes entering experimental huts. The purpose of these investigations was to determine the appropriate application rate of VECTRON™ T500 for field use and its efficacy against resist[1]ant mosquitoes in comparison to a WHO listed IRS product. Mortality following exposure to VECTRON™ T500 treated surfaces was assessed at 72 hours post-exposure due to the slower mode of action of broflanilide on the mosquitoes34. Clothianidin and chlorfenapyr, which also give delayed mortality with mosquitoes, are prequalified by WHO-PQT for use as an IRS products and an insecticide treated net for malaria vector control ^13,35,36^* **could be deleted. **
3. The following text
  Other studies have shown residual efficacy of Actellic® 300CS up to six months ^38^. Our data corroborate these studies.
  To be rephrased as follows:
  Residual efficacy of Actellic® 300CS is in the present study is similar to the findings of a previous study ^38^

**Conclusion**

1. The first sentence to be deleted
2. The third and fourth sentences to be combined as follows:
  The present study has defined the dose for VECTRON™ T500 to be used in Community trials i.e. 100 mg a.i./m2

**Grammatical and typing /spelling errors**

 There are few typing errors. For example:

- Pourcentage instead of Percentage (table 3)
- Resistance instead of resistant (page 12)
- resistant instead of resistance (abstract)

I find the manuscript as the one that requires minor corrections before it can be considered for indexing in *Gates Open Research*

 Sincerely,



 Dr. Johnson Matowo (Ph.D)

Is the work clearly and accurately presented and does it cite the current literature?

Yes

If applicable, is the statistical analysis and its interpretation appropriate?

Yes

Are all the source data underlying the results available to ensure full reproducibility?

Yes

Is the study design appropriate and is the work technically sound?

Yes

Are the conclusions drawn adequately supported by the results?

Yes

Are sufficient details of methods and analysis provided to allow replication by others?

Yes

Reviewer Expertise:

Medical entomology, with focus on understanding of the Biochemical and Molecular Basis of Insecticide Resistance in Malaria Vectors.

I confirm that I have read this submission and believe that I have an appropriate level of expertise to confirm that it is of an acceptable scientific standard.

# Response

**Published:** 2022-06-18

**Authors:** koama bayili

**Affiliations:** Institut de recherche en sciences de la santé, Burkina Faso

Dear Dr. Johnson Matowo

 We thank  Dr. Johnson Matowo for his constructive comments and suggestions to improve the manuscript. The manuscript will be revised accordingly. All changes or additions in the text will be done in the revised version before submit it. All the changes will be in yellow in the revised version.

 Thank you very much again for your time in reviwing this manuscript.
